# Hypertension and chronic kidney disease affect long-term outcomes in patients with stable coronary artery disease receiving percutaneous coronary intervention

**DOI:** 10.1038/s41598-018-35982-4

**Published:** 2018-12-05

**Authors:** Mao-Jen Lin, Wen-Chieh Yang, Chun-Yu Chen, Chia-Chen Huang, Hsun-Yang Chuang, Feng-Xia Gao, Han-Ping Wu

**Affiliations:** 1 Division of Cardiology, Department of Medicine, Taichung Tzu Chi Hospital, The Buddhist Tzu Chi Medical foundation, Taichung, Taiwan; 2 Department of Medicine, School of Medicine, Tzu Chi University, Hualien, Taiwan; 3 Department of Pediatric Emergency Medicine, China Medical University Children’s Hospital, China Medical University, Taichung, Taiwan; 4 Department of Medicine, College of Medicine, China Medical University, Taichung, Taiwan; 5 Department of Public Health, Chung Shan Medical University, Taichung, Taiwan; 6 Department of Research, Taichung Tzu Chi Hospital, Buddhist Tzu Chi Medical Foundation, Taichung, Taiwan; 7 Department of Medical Research, China Medical University Children’s Hospital, China Medical University, Taichung, Taiwan

## Abstract

Percutaneous coronary intervention (PCI) is commonly used for patients with coronary artery disease (CAD). However, the effects of chronic kidney disease (CKD) and hypertension (HT) on long-term outcomes in patients with stable CAD receiving PCI are still unclear. A total of 1,676 patients treated with PCI were prospectively enrolled and divided into 4 groups according to the presence or absence of HT or CKD. General characteristics, clinical medications, risk factors, angiographic findings, and long-term outcomes were analyzed. Patients with CKD had the highest rate of all-cause and cardiovascular (CV) mortality (both *P* < 0.01). Patients with CKD alone had the lowest event-free rate of all-cause and CV deaths (both *P* < 0.001). Based on Cox proportional hazard model, patients with CKD alone had the highest risk of all-cause death (HR:2.86, 95% CI:1.73–4.75) and CV death (HR: 3.57,95% CI:2.01–6.33); while patients with both CKD and HT had the highest risk of repeat PCI (HR: 1.42, 95% CI:1.09–1.85).We found that in stable CAD patients after undergoing PCI, those with CKD alone had the highest long-term mortality. Comorbid CKD appears to increase risk in patient with HT, whereas comorbid HT doesn’t seem to increase risk in patients with CKD.

## Introduction

Percutaneous coronary intervention (PCI) is a common and important therapeutic strategy for treating patients with coronary artery disease (CAD). Nevertheless, several risk factors affect long-term outcome after PCI. Diabetes mellitus (DM) and hypertension (HT) are significant risk factors that affect outcomes in CAD patients undergoing PCI^1–3^. Recently, chronic kidney disease (CKD) has been regarded as a new risk factor associated with outcomes in patients undergoing PCI^4–6^.

For patients who develop acute coronary syndrome (ACS) after receiving PCI, HT does not appear to affect short-term and long-term outcomes^7,8^. However, the presence of CKD does adversely affect patients with ACS receiving PCI^9,10^. On the other hand, the influence of CKD and HT on patients with stable CAD receiving PCI is less clear. The impact of DM and HT on long-term outcomes in patients with stable CAD after receiving PCI has also been well studied^11^. Comorbid HT does not seem to increase risk in diabetic patients, whereas comorbidity with DM does increase risk in hypertensive patients. In contrast, in a recent study, DM and CKD had an additive effect on adverse long-term outcomes in patients receiving PCI. The presence of CKD seemed to predict a poorer outcome than did DM^12^.

The isolated and combined effect of HT and CKD on long-term prognosis in patients with stable CAD undergoing PCI remains unclear. We designed a study to validate and compare long-term outcomes among 4 groups of patients: (1) patients without HT and CKD (control group), (2) patients with HT alone, (3) patients with CKD alone, and (4) patients with both HT and CKD. In addition, we analyzed the adverse predictors of clinical outcomes among these 4 groups.

## Methods

### Study Population

Using a prospective cohort design, we recruited male and female patients 20 to 90 years of age who were consecutively admitted to undergo PCI from the inpatient clinic at Taichung Tzu Chi Hospital, Taiwan, from June 2007 through December 2015. According to isolated or combined risk factors, the patients were divided into 4 groups: patients without HT or CKD (control group), patients with HT alone (HT group), patients with CKD alone (CKD group), and patients with both HT and CKD (CKD and HT group). Patients scheduled to undergo a schedule PCI procedure and who had a previous history of malignancy were excluded. Most patients received regular follow-up in the outpatient department (OPD). For some patients who were lost to follow-up, a telephone call was used to contact the patients or their families. At the end of the study, a full survey of cardiovascular (CV) mortality mortality, all-cause mortality, myocardial infarction (MI), and repeated PCI procedures was completed. The study protocol was approved by the Institution Review Board and ethics committee of Taichung Tzu Chi Hospital, Taiwan (REC106-10) and written informed consent was obtained from all participants. This cohort study also fulfilled the guidance of Strengthening the Reporting of Observational Studies in Epidemiology (STROBE) statenment^13^.

### Data processing and analysis

General demographic data, including body habitus, biochemical data, angiographic findings from cardiac catheterization, exposure to risk factors, and use of different therapeutic strategies, such as medications and invasive procedures (balloon angioplasty, bare metal stent deployment, or drug-eluting stent deployment) were all collected. Hypertension is defined as a ususal blood pressure (BP) of 140/90 mm Hg or higher, BP levels for which the benefits of pharmacologic treatment have been definitely established^14^. Diabetes was defined as a fasting plasma glucose level >126 mg/dL, a casual plasma glucose level >200 mg/dL, or a hemoglobin A1c (HbA1c) level >6.5%^15^. Estimated glomerular filtration rate (eGFR) and stage of chronic kidney disease (CKD) were established. CKD was divided into 5 stages: stage l, eGFR ≥90 mL/min/1.73 m;^2^ stage 2, eGFR, 60–89 mL/min/1.73 m;^2^ stage 3, eGFR, 30–59 mL/min/1.73 m;^2^ stage 4, eGFR, 15–29 mL/min/1.73 m;^2^ and stage 5, eGFR <15 mL/min/1.73 m^2^ or dialysis. CKD was defined as an eGFR <60 mL/min/1.73 m^2^, which was equal to or greater than CKD stage 3, in the current study^16^. Hypercholesterolemia was defined as a serum cholesterol level >200 mg/dL or a low-density lipoprotein-cholesterol (LDL-C) level >100 mg/dL. For the angiographic and hemodynamic data, we measured the central aortic pressure (CAP) and left ventricular ejection fraction (LVEF). CAP was obtained by using a pigtail catheter to directly measure ascending aortic pressure. Angiographic findings, including the number of diseased vessels and lesion locations, were calculated, and lesion severity and complexity were evaluated via the synergy between PCI with Taxus Express paclitaxel-eluting stent (Boston Scientific, Marlborough, MA, USA) and cardiac surgery score (SYNTAX score)^17^. LVEF was estimated through angiographic ventriculography or stress ventriculography. MI was defined as an MI attack after index PCI, accompanied by a 3-fold elevation of cardiac enzymes from the baseline value. General characteristics, major exposure to risk factors, angiographic findings, and PCI strategies were analyzed. The primary endpoints, including all-cause mortality, CV mortality, MI and repeated PCI, were also compared among the 4 groups. CV mortality was defined as death resulting from an acute MI, sudden cardiac death, and death due to heart failure (HF), due to stroke, due to CV procedures, due to CV hemorrhage, and due to other CV causes. Repeated PCI was defined as repeated percutanous coronary intervention after discharge of index PCI procedure if clinically indicated. Follow-up began at the time of the index PCI procedure, and continued through December 31, 2016, or until any of the previously mentioned primary endpoints occurred.

### Statistical analysis

Statistical analysis was primarily used to assess variance among the 4 groups. Analysis of variance (ANOVA) was used to test continuous variables, and the chi-square test was utilized to test categorical variables. Log-rank test and Kaplan–Meier curves were applied to compare survival differences. Cox proportional hazards model was used to test the effect of independent variables on hazards. Stepwise regression and signficant variables in ANOVA and Chi-square test were put into Cox prorotional hazards model to analyze each outcomes. *P-*values < 0.05 were considered significant. All analyses were performed using the statistical package SPSS for Windows (Version 23.0, SPSS Inc., Chicago, IL, USA).

### Ethics approval and consent to participate

The study protocol was approved by the Institution Review Board and ethics committee of Taichung Tzu Chi Hospital, Taiwan (REC106-10), and written informed consent was obtained from all study participants.

## Results

A total of 1,676 patients who had undergone a successful PCI procedure were enrolled during the study period. Among the 4 study groups, 434 patients were in the control group, 474 patients had HT alone, 246 patients had CKD alone, and 522 patients had both HT and CKD. The CKD group, and those with both HT and CKD had shorter mean follow-up times (control group: 51.3 ± 26.5 months; HT group, 50.0 ± 25.8 months; CKD group, 38.4 ± 25.9 months; and the group with both HT and CKD, 39.9 ± 23.8 months; *P* < 0.01).

General patient characteristics are listed in Table 1. Patients with CKD alone and with both HT and CKD were much older than were patients in the other groups (*P* < 0.01). There were no significant differences in body mass index (BMI) among the 4 patient groups (*P* = 0.87). As for hemodynamic parameters, patients with both HT and CKD had the highest central systolic pressure (CSP) and highest central pulse pressure (CPP) compared to those in the other groups (*P* < 0.01). In terms of baseline biochemistry, patients with CKD alone had the lowest cholesterol and LDL-C levels (both *P* < 0.01).

**Table 1:** General characteristics of the study population.

	Study groups				*P*-value
	Control (n = 434)	HT alone (n = 474)	CKD alone (n = 246)	HT and CKD (N = 522)	
Age (years)	57.9 ± 10.5	59.4 ± 10.7	70.3 ± 9.4	70.5 ± 10.8	<0.01*
Weight (kg)	69.5 ± 11.6	72.2 ± 13.6	62.3 ± 11.6	64.4 ± 12.0	<0.01*
Height (cm)	1.64 ± 0.08	1.64 ± 0.09	1.60 ± 0.08	1.60 ± 0.08	<0.01*
BMI (kg/m^2^)	25.7 ± 3.6	26.8 ± 3.9	24.1 ± 3.5	25.1 ± 4.1	<0.01*
CSP (mmHg)	126.1 ± 18.8	140.0 ± 22.1	128.8 ± 24.5	144.9 ± 26.2	<0.01*
CDP (mmHg)	72.9 ± 11.4	76.7 ± 13.6	67.1 ± 12.6	71.5 ± 13.6	0.33
CPP (mmHg)	53.2 ± 15.4	63.3 ± 18.8	61.7 ± 20.6	73.3 ± 23.3	<0.001*
Cholesterol (mg/dL)	181.9 ± 44.4	181.2 ± 42.8	169.2 ± 40.8	172.5 ± 44.7	0.02*
HDL (mg/dL)	39.8 ± 15.6	37.5 ± 15.1	40.3 ± 15.8	39.8 ± 16.8	0.59
TG (mg/dL)	152.4 ± 102.1	167.9 ± 113.4	141.1 ± 109.2	149.8 ± 98.0	0.62
LDL (mg/dL)	111.6 ± 38.8	109.9 ± 37.4	100.6 ± 34.9	102.7 ± 37.7	<0.01*
Serum creatinine (mg/dL)	1.0 ± 0.2	1.0 ± 0.3	2.5 ± 2.6	2.9 ± 3.1	<0.01*

HT alone: hypertension alone. CKD alone: chronic kidney disease alone. HT and CKD: both hypertension and chronic kidney disease. BMI: body mass index. CSP: central aortic systolic pressure. CDP: central aortic diastolic pressure. CPP: central pulse pressure. HDL: high-density lipoprotein cholesterol. LDL: low-density lipoprotein cholesterol. TG: triglyceride.

*Significant.

Demographic data for the study population are shown in Table 2. Female gender, diabetes preponderance, and the highest rate of previous stroke history were observed in patients with HT and CKD (all *P* < 0.01). However, patients with CKD alone had the lowest prevalence of hypercholesterolemia and the highest rate of previous MI (both *P* < 0.01). When we evaluated medications prescribed after PCI, we found that patients with CKD alone used the least aspirin (*P* < 0.01) and the most diuretics (*P* < 0.01) of the 4 groups. This group also had the lowest usage of beta-blockers (BB), calcium channel blockers (CCB), and statins (all *P* < 0.01).

**Table 2:** Demographics of the study population, and medications prescribed after index PCI.

	Study groups				*P*-value
	Control (n = 434)	HT alone (n = 474)	CKD alone (n = 246)	HT and CKD (n = 522)	
Gender					<0.01*
Female	69 (15.9%)	112 (23.6%)	62 (25.2%)	205 (39.3%)	
Male	365 (84.1%)	362 (76.4%)	184 (74.8%)	317 (60.7%)	
Dyslipidemia					0.02*
No	109 (25.1%)	121 (25.5%)	81 (32.9%)	166 (31.2%)	
Yes	325 (74.9%)	353 (74.5%)	165 (67.1%)	356 (68.2%)	
DM history					<0.001*
No	307 (70.7%)	290 (61.2%)	139 (56.1%)	244 (46.8%)	
Yes	127 (29.3%)	184 (38.8%)	107 (43.9%)	278 (53.2%)	
Current smoker					<0.01*
No	239 (55.1%)	271 (57.2%)	164 (66.7%)	370 (70.9%)	
Yes	195 (44.9%)	203 (42.8%)	82(33.3%)	152 (29.1%)	
Previous MI					<0.01*
No	264 (60.8%)	346 (73.0%)	131 (53.3%)	343 (65.7%)	
Yes	170 (39.2%)	128 (27.0%)	115 (46.8%)	179 (34.3%)	
Stroke history					<0.01*
No	421 (97.0%)	448 (94.5%)	228 (92.7%)	474 (90.8%)	
Yes	13 (3.0%)	26 (5.5%)	18 (7.3%)	48 (9.2%)	
CABG history					0.24
No	432 (99.5%)	472 (99.6%)	245 (99.6%)	515 (98.7%)	
Yes	2 (0.5%)	2 (0.4%)	1 (0.4%)	7 (1.3%)	
Aspirin					<0.01*
No	25 (5.8%)	31 (6.5%)	36 (14.6%)	62 (11.9%)	
Yes	409 (94.2%)	443 (93.5%)	210 (85.4%)	460 (88.1%)	
P2Y12 inhibitor					0.56
No	78 (18%)	77(16.2%)	37(15.0%)	77 (14.7%)	
Yes	356 (82%)	397 (83.8%)	209 (85.0%)	445 (85.3%)	
Diuretics					<0.01*
No	366 (84.3%)	379 (80.0%)	175 (71.1%)	392 (75.1%)	
Yes	68 (15.7%)	95 (20.0%)	71 (28.9%)	130 (24.9%)	
BB					<0.01*
No	250 (57.6%)	242 (51.0%)	157 (63.8%)	276 (52.9%)	
Yes	184 (42.4%)	232 (49.0%)	89 (36.2%)	246 (47.1%)	
CCB					<0.01*
No	330 (76.0%)	278 (58.6%)	192 (78.0%)	333 (63.8%)	
Yes	104 (24.0%)	196 (41.4%)	54 (22.0%)	189 (36.2%)	
ACEI					<0.01*
No	319 (73.5%)	390 (82.3%)	184 (74.8%)	439 (84.1%)	
Yes	115 (26.5%)	84 (17.7%)	62 (25.2%)	83 (15.9%)	
ARB					<0.01*
No	387 (89.2%)	302 (63.7%)	211 (85.8%)	346 (66.3%)	
Yes	47 (10.8%)	172 (36.3%)	35 (14.2%)	176 (33.7%)	
Statin					<0.01*
No	269 (62.0%)	252 (53.2%)	194 (78.9%)	357 (68.4%)	
Yes	165 (38.0%)	222 (46.8%)	52 (21.1%)	165 (31.6%)	
Fibrate					0.04*
No	408 (94.0%)	432 (91.1%)	237 (96.3%)	497 (95.2%)	
yes	26 (6.0%)	42 (8.9%)	9 (3.7%)	25 (4.8%)	

HT alone: hypertension alone. CKD alone: chronic kidney disease alone. HT and CKD: both hypertension and chronic kidney disease. Previous MI: history of previous myocardial infarction. CABG history: history of coronary artery bypass graft. P2Y12 inhibitor: P2Y12 receptor inhibitor of platelet. BB: beta-blockers. CCB: calcium channel blocker. ACEI: angiotensin-converting enzyme inhibitor. ARB: angiotensin receptor blocker.

*Significant.

Angiographic findings and clinical outcomes are shown in Table 3. Based on angiographic results, multi-vessel disease was found more frequently in patients with both CKD alone and those with both HT and CKD (*P* < 0.01). Patients with both HT and CKD also had the highest usage of bare metal stent deployment (*P* < 0.01).

**Table 3:** Demography of angiographic findings and outcome.

Variable	Study groups				*P*-value
	Control (n = 434)	HT alone (n = 474)	CKD alone (n = 246)	HT and CKD (n = 522)	
Follow-up time (months)	51.3 ± 26.5	50.0 ± 25.8	38.4 ± 25.9	39.9 ± 23.8	<0.01*
Number of diseased vessels					<0.01*
Single-vessel disease	249 (57.4%)	242 (51.1%)	107 (43.5%)	196 (37.5%)	
Dual-vessel disease	118 (27.2%)	131 (27.6%)	70 (28.5%)	181 (34.7%)	
Triple-vessel disease	67 (15.4%)	101 (21.3%)	69 (28.0%)	145 (27.8%)	
Mean of treated vessels	1.2 ± 0.4	1.3 ± 0.5	1.2 ± 0.4	1.4 ± 0.6	<0.01*
Mean of treated lesions	1.4 ± 0.6	1.5 ± 0.8	1.4 ± 0.8	1.7 ± 0.9	<0.01*
Type of intervention Balloon angioplasty	142 (32.7%)	137 (28.9%)	88 (35.8%)	178 (34.1%)	0.20
BMS deployment	152 (35.0%)	194 (40.9%)	97 (39.4%)	248 (47.5%)	<0.01*
DES deployment	175 (40.3%)	200 (42.2%)	84 (34.2%)	193 (37.0%)	0.15
SYNTAX score	11.0 ± 8.1	10.5 ± 7.6	11.6 ± 8.3	11.3 ± 8.0	0.09
LVEF	0.6 ± 0.1	0.6 ± 0.1	0.6 ± 0.2	0.6 ± 0.1	0.06
MI					0.48
Yes	18 (4.2%)	14 (3.0%)	13 (5.3%)	21 (4.0%)	
No	416 (95.8%)	460 (97.0%)	233 (94.7%)	501 (96.0%)	
CV death					<0.01*
Yes	18 (4.2%)	12 (2.5%)	39 (15.9%)	50 (9.6%)	
No	416 (95.9%)	462 (97.5%)	207 (84.2%)	472 (90.4%)	
All-cause death					<0.01*
Yes	27 (6.2%)	17 (3.6%)	72 (29.3%)	79 (15.1%)	
No	407 (93.8%)	457 (96.4%)	174 (70.7%)	443(84.9%)	
Re-PCI					0.04*
Yes	129 (29.7%)	103 (21.7%)	59 (24.0%)	128 (24.5%)	
No	305 (70.3%)	371 (78.3%)	187 (76.0%)	394 (75.5%)	

BMS: bare metal stent. DES: drug-eluting stent. LAD: left anterior descending artery. Lcx: left circumflex artery. RCA: right coronary artery. SYNTAX score: Synergy between Percutaneous Coronary Intervention with Taxus and Cardiac Surgery score. LVEF: left ventricular ejection fraction. MI: myocardial infarction. Re-PCI: repeated percutaneous coronary intervention.

^*^Significant.

**Figure 1:**
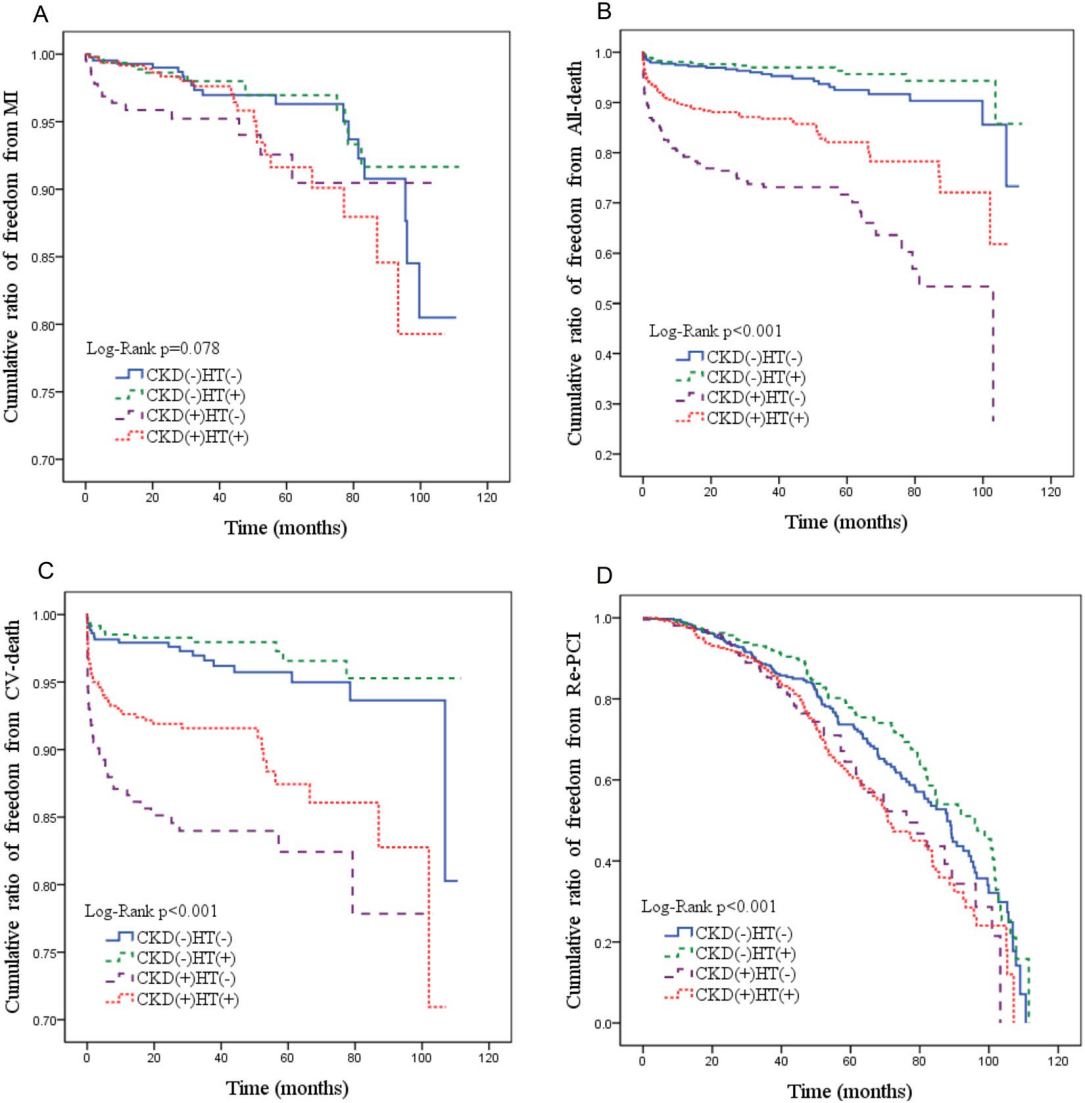
(**A**) Cumulative ratio of freedom from MI among the 4 patient groups (*P* = 0.078). (**B**) Cumulative ratio of freedom from all-death causes among the 4 groups (*P* < 0.001). (**C**) Cumulative ratio of freedom from CV deaths among the 4 groups (*P* < 0.001). (**D**) Cumulative ratio of freedom from re-PCI among the 4 groups (*P* < 0.001).

As for adverse outcomes, patients with CKD alone had the highest all-cause mortality (CKD group: 72/246, control group: 27/434. HT group:17/474, HT and CKD group:79/522, P < 0.01) and the highest CV mortality (CKD group:39/246, control group: 18/434, HT group:12/474, HT and CKD group: 50/522, P < 0.01); however, there was no difference in the rate of MI among the 4 groups (control group: 18/434, HT group:14/474, CKD group:13/246, HT and CKD group: 21/522, P = 0.48). Figure 1 reveals the cumulative rate of freedom from MI, cardiovascular death, all-cause death, and repeated PCI among the 4 groups. Patients with CKD alone had the lowest event-free rate of all-cause mortality and CV mortality (both *P* < 0.001).

Table 4 lists the related outcome predictors for MI, all-cause death, CV death, and repeated PCI, based on outcome analysis using the Cox proportion hazard model. Compared with the control group, patients with CKD alone had the greatest risk of CV mortality, all-cause mortality (hazard ratio [HR]: 2.86, and 3.57, respectively; both *P* < 0.01). Patients with both CKD and HT had the highest rate of repeated PCI (HR = 1.42). In addition, we found that previous MI and SYNTAX scores were both predictors for future MIs (HR: 2.41 and 1.04, respectively. Statin use reduced the risk of MI (HR: 0.47). Age, previous MI, a history of stroke, and SYNTAX score were all predictors for all-cause mortality (HR: 1.04, 2.88, 1.79, and 1.03, respectively). BB and statin usage reduced the risk of death (HR: 0.62 and 0.39, respectively). Previous MI and SYNTAX score were both predictors for CV mortality (HR: 3.81 and 1.04, respectively); use of BB, angiotensin-converting enzyme inhibitors (ACEI) and statins reduced the risk of CV mortality (HR: 0.52, 0.43, 0.40, and 0.53, respectively). Finally, cigarette smoking, BB usage, and SYNTAX score were all associated with repeated PCI procedures (HR: 1.52, 1.27, and 1.02, respectively). The hazard ratio interval estimation method was used to determine whether the confounding variable exist, and we found that age, vessel number, use of aspirin and use of statin were not confounding variables in this study.

**Table 4:** Significant predictors of outcome based on the Cox proportion hazard model for MI, all-death, CV-death, and repeated PCI.

Variables	MI^a^	All-death^b^	CV-death^c^	Repeated PCI^d^
	HR^a^ (95% CI)	HR^a^ (95% CI)	HR^a^ (95% CI)	HR^a^ (95% CI)
Group				
Control	1.00 (Reference)	1.00 (Reference)	1.00 (Reference)	1.00 (Reference)
HT alone	0.92 (0.45–1.87)	0.57 (0.29–1.15)	0.76 (0.36–1.58)	0.80 (0.61–1.03)
CKD alone	1.60 (0.77–3.30)	2.86 (1.73–4.75)**	3.57 (2.01–6.33)**	1.35 (0.97–1.87)
HT and CKD	1.49 (0.78–2.84)	1.61 (0.96–2.70)	2.51 (1.45–4.35)**	1.42 (1.09–1.85)**
Age	**—**	1.04 (1.02–1.05)**	**—**	**—**
Smoking	**—**	**—**	**—**	1.52 (1.25–1.86)**
Previous MI	2.41 (1.44–4.05)**	2.88 (2.04–4.08)**	3.81 (2.53–5.75)**	**—**
Stroke history	1.41 (0.56–3.57)	1.79 (1.07–3.00) ^*^		**—**
Diuretics	**—**	**—**	1.14 (0.76–1.73)	**—**
DES	**—**	**—**	**—**	**—**
Aspirin	1.20 (0.47–3.04)	**—**	1.19 (0.66–2.14)	**—**
P2Y12 inhibitor	1.29 (0.60–2.75)	**—**	1.76 (0.88–3.50)	**—**
BB	**—**	0.62 (0.43–0.87)**	0.52 (0.35–0.77)**	1.27 (1.04–1.54)*
CCB	**—**	**—**	**—**	**—**
ACEI	**—**	**—**	0.48 (0.30–0.77)**	**—**
ARB	**—**	**—**	**—**	**—**
Statin	0.47 (0.26–0.82)**	0.39 (0.25–0.60)**	0.53 (0.34–0.83)**	**—**
SYNTAX	1.04 (1.01–1.06)**	1.03 (1.02–1.05)**	1.04 (1.02–1.06)**	1.02 (1.01–1.03)**

HT: hypertension. CKD: chronic kidney disease. HT and CKD: both hypertension and CKD. Previous MI:

Previous history of myocardial infarction. DES: drug-eluting stent. P2Y12 inhibitor: P2Y12 receptor

inhibitor of platelet. BB: beta-blockers. CCB: calcium channel blocker. ACEI: angiotensin -converting

enzyme inhibitor. ARB: angiotensin receptor blocker. SYNTAX score: Synergy between Percutaneous

Coronary Intervention with Taxus and Cardiac Surgery score.

*P < 0.05, **P < 0.01.

^a^MI model: y = βdummyDH1 + βdummyDH2 + βdummyDH3 + βMI + βstroke + βstatin + βsyntax.

^b^All-death model: y = βdummyDH1 + βdummyDH2 + βdummyDH3 + βage + βCKD + βMI + βstroke + βbetab + βstatin + βsyntax.

^c^CV-death model: y = βdummyDH1 + βdummyDH2 + βdummyDH3 + βMI + βstroke + βdiuretics + βbetab + βACEI + βstatin + βsyntax.

^d^Repeated PCI model: y = βdummyDH1 + βdummyDH2 + βdummyDH3 + βMI + βsmoking + βbetab + βsyntax.

## Discussion

Among patients with stable CAD who had PCI, those with CKD alone had the highest rate of all-cause mortality and CV mortality compared with patients without CKD and HT, patients with CKD and HT, and patients with HT alone. Patients with both CKD and HT had the highest rate of repeated PCI procedures; however there was no difference in MI rate among the 4 patient groups. Previous MI history and SYNTAX score were predictors for MI. Age, previous MI, stroke history, and SYNTAX scores were predictors for all-cause death. Previous MI and SYNTAX score were both predictors for CV death. Smoking and use of BB increased the risk of repeated PCI. On the other hand, use of statins could reduce the risk of a MI. Usage of BB and statin could reduce all-cause mortality; whereas use of BB, ACEI, and statins could reduce the risk of CV mortality.

In our study, patients with CKD alone, along with patients with both HT and CKD, were older and had lower body weights; these findings are compatible with the results of a previous study^18^. Patients with both HT and CKD had more elevated central pulse pressure (CPP) than did patients with HT alone, implying that CKD had an additive effect on progression of arterial stiffness in patients with HT. Elevated CPP is strongly associated with adverse cardiovascular outcomes in patients after undergoing a PCI procedure, as shown by results of a previous study^19,20^. On the other hand, compared to patients with CKD alone, patients with both HT and CKD had higher serum creatinine levels. Hence, hypertension may have an adverse impact on renal function in patients with CKD^21^.

Patients with both HT and CKD had a higher prevalence rate of diabetes than did patients with isolated HT or isolated CKD. Although it has been reported that either CKD or HT may increase insulin resistance and the incidence of developing metabolic syndrome^22–24^, it isn’t clear whether HT and CKD had an additive effect on insulin resistance or development of the metabolic syndrome. In contrast, compared to patients with HT alone or patients with both HT and CKD, patients with isolated CKD had the highest prevalence of an MI history and the least use of statins and BB. Cardiovascular drugs, including BB and statins, could improve long-term mortality in patients after MI^25^. Even when the serum LDL level is <70 mg/dL, statin use could improve cardiovascular outcomes in CAD patients after ACS^26^. It is likely that underuse of statins may be due to the fact that CKD patients had the lowest cholesterol and LDL-C levels, and clinical practice in this study was based on early lipid treatment guidelines^27^.

There were no differences in type of intervention, such as balloon angioplasty or drug-eluting stent deployment among the patients, but bare-metal stent deployment was used more frequently in patients with both HT and CKD. In terms of the number of diseased vessels, patients with both HT and CKD had the highest prevalence of double-vessel disease, while patients with CKD alone had the highest prevalence of triple-vessel disease. Compared with patients with HT alone, patients with both HT and CKD had a significantly higher risk of developing multi-vessel disease. However, when compared with patients with CKD alone, patients with both HT and CKD did not have a significantly increased risk of developing multi-vessel disease. Comorbidity with CKD in HT patients might increase the risk of developing multi-vessel disease compared to patients with HT alone. In contrast to the results of a previous study, the combination of DM and CKD seemed to have an additive effect on the progression of atherosclerosis and the development of multi-vessel disease. This was not the case among patients with DM or CKD alone. On the other hand, in terms of the mean number of treated vessels and lesions, patients with CKD alone had the fewest treated vessels and lesions. This may have been due to the less-aggressive invasive strategy that was chosen when there was concern about the possible effects of contrast-induced nephropathy (CI N). Therefore, the dominance of triple-vessel disease and a strategy of selecting a less aggressive approach may affect long-term outcomes in patients with CKD alone after undergoing PCI.

In our study, patients with CKD alone had the highest rate of all-cause mortality and CV mortality. Patients with CKD alone had a higher rate of previous MI and having triple-vessel disease, coupled with a conservative invasive strategy, and less statin use. Compared to the CKD group, patients with both CKD and HT had a lower rate of all-cause mortality and CV mortality. However, compared to the HT group, patients with both CKD and HT did have a higher rate of all-cause mortality and CV mortality after undergoing PCI. Although patients with both CKD and HT had more mean treated vessels and lesions, they also had a higher prevalence of multi-vessel disease, implying that CKD may pose an additive effect on the progression of atherosclerosis and may also cancel any benefits of an aggressive intervention strategy.

### Study limitations

Our study did have some limitations. First, we did not fully survey the patients’ adherence to medication and the dosage of medications, such as using strict blood glucose control or blood pressure control. Second, functional evaluations of the atherosclerotic lesions and plaque composition analysis, such as fraction flow reserve (FFR) measurements or intravascular ultrasound (IVUS), were not used in this study. This may have had an impact on the decision-making about who was enrolled in index PCI studies. It has been reported that CKD was an independent predictors for FFR measurement; besides, positive findings of FFR were lower in patients with CKD^28^. Third, since the number of patients in the CKD alone group was smaller than that of the other groups, the power of this study may have been affected.

## Conclusions

In stable CAD patients undergoing PCI, patients with CKD alone had the highest long-term mortality rate after undergoing PCI compared with patients without HT and CKD and with those with both HT and CKD or isolated HT. Comorbidity with HT appeared not to increase risk in CKD patients, whereas comorbidity with CKD did increase risk in HT patients.

## Data Availability

The data that support the findings of this study are available, on reasonable request, from the corresponding author.
